# Rheumatoid Purpura in Children: A Retrospective Study

**DOI:** 10.7759/cureus.73443

**Published:** 2024-11-11

**Authors:** Hajar Boudarbala, Aziza Elouali, Anane Sara, Maria Rkain, Abdeladim Babakhouya

**Affiliations:** 1 Department of Pediatrics and Child Health, Mohammed VI University Hospital, Oujda, MAR; 2 Faculty of Medicine and Pharmacy, Mohammed I University, Centre Hospitalier Universitaire (CHU) Mohammed VI, Oujda, MAR

**Keywords:** arthralgia, child, digestive complication, nephropathy, rheumatoid purpura

## Abstract

Introduction

Rheumatoid purpura, also referred to as Schönlein-Henoch disease, is a form of systemic vasculitis that is characterized by the presence of IgA deposits within the walls of small vessels. The primary symptoms include skin purpura, arthralgia, abdominal discomfort, and urinary tract abnormalities. While often benign, the disease can result in severe complications, particularly those affecting the digestive and renal systems, which require urgent medical attention.

Objectives

This study aims to analyze the epidemiological, clinical, biological, therapeutic, and evolutionary characteristics of rheumatoid purpura in order to enhance understanding of its clinical and therapeutic management. The aim is to expand knowledge of the disease and optimize treatment strategies to prevent serious complications.

Methods and results

This retrospective study encompasses 39 cases of rheumatoid purpura treated at the General Pediatrics Department of CHU Mohamed VI in Oujda, Morocco, over a period of nine years and six months from January 2015 to June 2024. The patients had a mean age of 6.5 years, and there was a marked male predominance (sex ratio of 2.57). All patients exhibited cutaneous purpura (100%). Approximately 75% of patients exhibited joint involvement, 89% of cases were associated with digestive manifestations, and 28% of cases demonstrated renal involvement.

Conclusion

Rheumatoid purpura is a pediatric condition often triggered by an upper respiratory infection, characterized by IgA deposits. While typically benign, it can lead to severe complications, particularly in the digestive and renal systems. Corticosteroids are key in managing acute cases, although their preventive efficacy is limited. Early and appropriate management is crucial for improving long-term outcomes.

## Introduction

Rheumatoid purpura (RP), or Schönlein-Henoch disease, is a dysimmune systemic vasculitis affecting small-caliber vessels [1]. Recent advances in understanding the disease's pathophysiology, particularly regarding IgA deposits in blood vessels and the underlying immunological mechanisms, have significantly improved clinical management strategies. The disease was initially delineated by Dr. Heberden in 1801 and subsequently refined by Dr. Henoch and Dr. Schönlein in 1837. This is the reason why, in some countries, it is referred to as Henoch-Schönlein purpura [2].

In the Moroccan context, RP remains a significant epidemiological concern due to the frequency of cases and its potential to cause severe complications, particularly digestive and renal. The disorder is most prevalent in children and adolescents, with a lower incidence in adults and a rare occurrence in infants. The clinical presentation of the disease is characterized by vascular purpura, which predominantly affects declivous areas. It is often accompanied by arthralgia or arthritis of the large joints, abdominal discomfort, and abnormalities in the urine sediment. It is a leukocytoclastic vasculitis characterized by the presence of IgA deposits, likely resulting from an autoimmune reaction triggered by various antigenic stimuli, particularly infectious [3,4].

In the majority of cases, RP is a benign condition. However, it can become a significant health concern due to the potential for digestive and renal complications, which can be life-threatening in the immediate and long term. The treatment of RP is primarily symptomatic, given the current lack of understanding regarding its etiopathogenesis. It is strongly suspected of involving an immunological mechanism, as evidenced by the presence of circulating immune complexes containing IgA.

The primary objective of this study is to analyze the epidemiological, clinical, biological, therapeutic, and evolutionary characteristics of this condition based on a series of cases observed in the General Pediatrics Department of the CHU Mohamed VI in Oujda, Morocco, over a period of nine and a half years (January 2015 to June 2024). Subsequently, we will compare our findings with existing literature in order to elucidate the distinctive characteristics of our series.

## Materials and methods

This is a retrospective study of RP conducted at the General Pediatrics Department of Mohammed VI University Hospital in Oujda, Morocco. The study encompasses 39 cases observed over a period of nine years and six months, from January 2015 to June 2024. The diagnosis of RP was confirmed based on the presence of cutaneous syndrome in conjunction with at least one of the three characteristic clinical signs: joint involvement, gastrointestinal symptoms, or renal abnormalities. Data were collected from patient records, including both paper and electronic formats, as well as from medical prescription forms, checkups, and hospital monitoring documents.

Several limitations were encountered in our study. Firstly, data collection proved difficult due to the incompleteness of certain files, making access to crucial information and clinical assessments sometimes uncertain. In addition, the non-informatization of old files hampered research, complicating the extraction and analysis of relevant data. Finally, patient follow-up also posed a problem, as several patients were lost to follow-up, limiting our ability to assess the evolution of their health status over the long term. These factors underline the need to improve medical data management for future studies.

## Results

Our study included 28 boys and 11 girls, resulting in a male-to-female ratio (M/F) of 2.57. Patients ranged in age from 3 to 15 years, with an average age of 6.5. The majority of patients (66%) were admitted to the hospital during the autumn and winter months.

**Figure 1:**
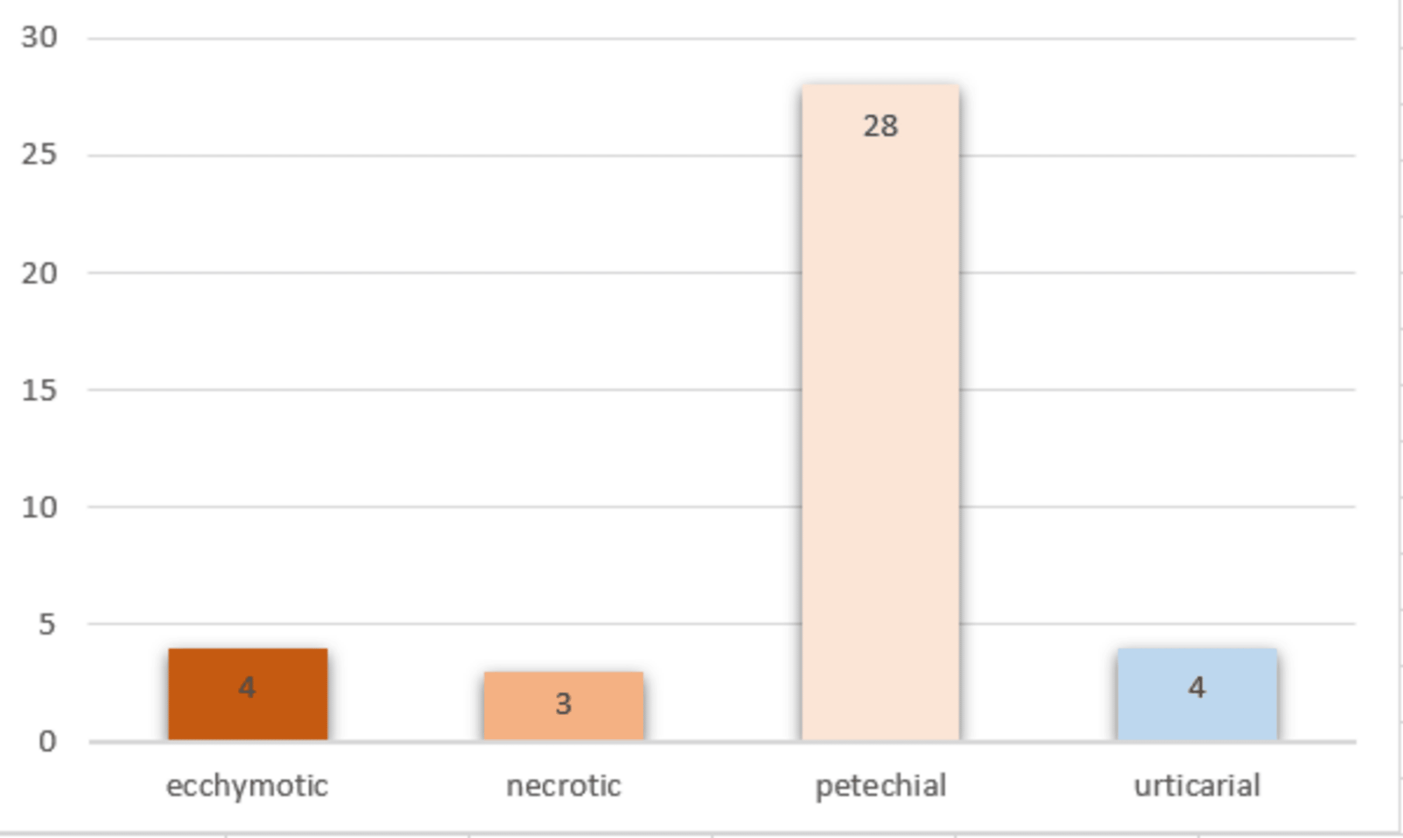
Distribution based on the appearance of purpura

The symptoms developed insidiously in 69% of patients, while 31% experienced a sudden onset. Purpura, the primary symptom, led to consultation in 100% of cases. It predominantly manifested as petechiae in 71% of cases, with ecchymosis and urticaria observed in 11% and necrosis in 7% (Figure 1). The purpura was bilateral, symmetrical, and mainly localized to the lower limbs in 100% of cases, with involvement of the upper limbs in 46% of cases. The purpura typically regressed within an average of 12 days, ranging from 6 to 30 days.

Joint involvement was present in 100% of cases. Arthralgia was common, primarily affecting the joints of the lower limbs (97%), with a particular predilection for the knees (97%). Only one case of arthritis was observed in our series. Digestive symptoms were present in 90% of patients, with abdominal pain in 89% and 10 lower gastrointestinal (GI) bleed in 30%; these symptoms could be either isolated or associated. There were 12 cases of digestive complications, including six instances of intestinal intussusception.

Of the 39 cases of RP, 11 patients (28%) exhibited renal involvement, comprising four girls and seven boys. Renal signs were predominantly biological, with clinical manifestations often being either absent or subtle (Table 1). Blood counts revealed thrombocytosis in 10 patients (26%); the blood count and the peripheral smear were normal for the rest.

**Table 1:** Manifestations of renal involvement in our series

Renal Signs	Number of Cases	Percentage
Microscopic hematuria	9	23%
Macroscopic hematuria	2	5%
Proteinuria	10	25%
Nephrotic syndrome	1	2%
Renal failure	0	0%
Hypertension	4	10%

Radiological examinations were conducted based on observed complications. An unprepared abdominal radiograph (UPR) was performed on two patients suspected of intestinal perforation, yielding normal results. An abdominal-renal ultrasound was carried out in 24 patients, revealing abnormalities in nine cases: three patients had evidence of ileocecal intussusception, while six patients had evidence of groin intussusception. Among the patients with renal signs, three underwent renal biopsy, which revealed lesions consistent with RP, including mesangial IgA deposits.

All patients were systematically admitted to the hospital, and analgesics and antispasmodics were prescribed. Oral corticosteroid therapy was administered to 24 patients (61% of cases). Among these, 17 patients received short-term treatment (approximately one month, followed by a gradual tapering of the dose) at a dose of 1 to 2 mg/kg/day, primarily for abdominal pain and renal impairment. Seven patients required three boluses of methylprednisolone, two of whom subsequently continued on oral corticosteroids.

**Figure 2:**
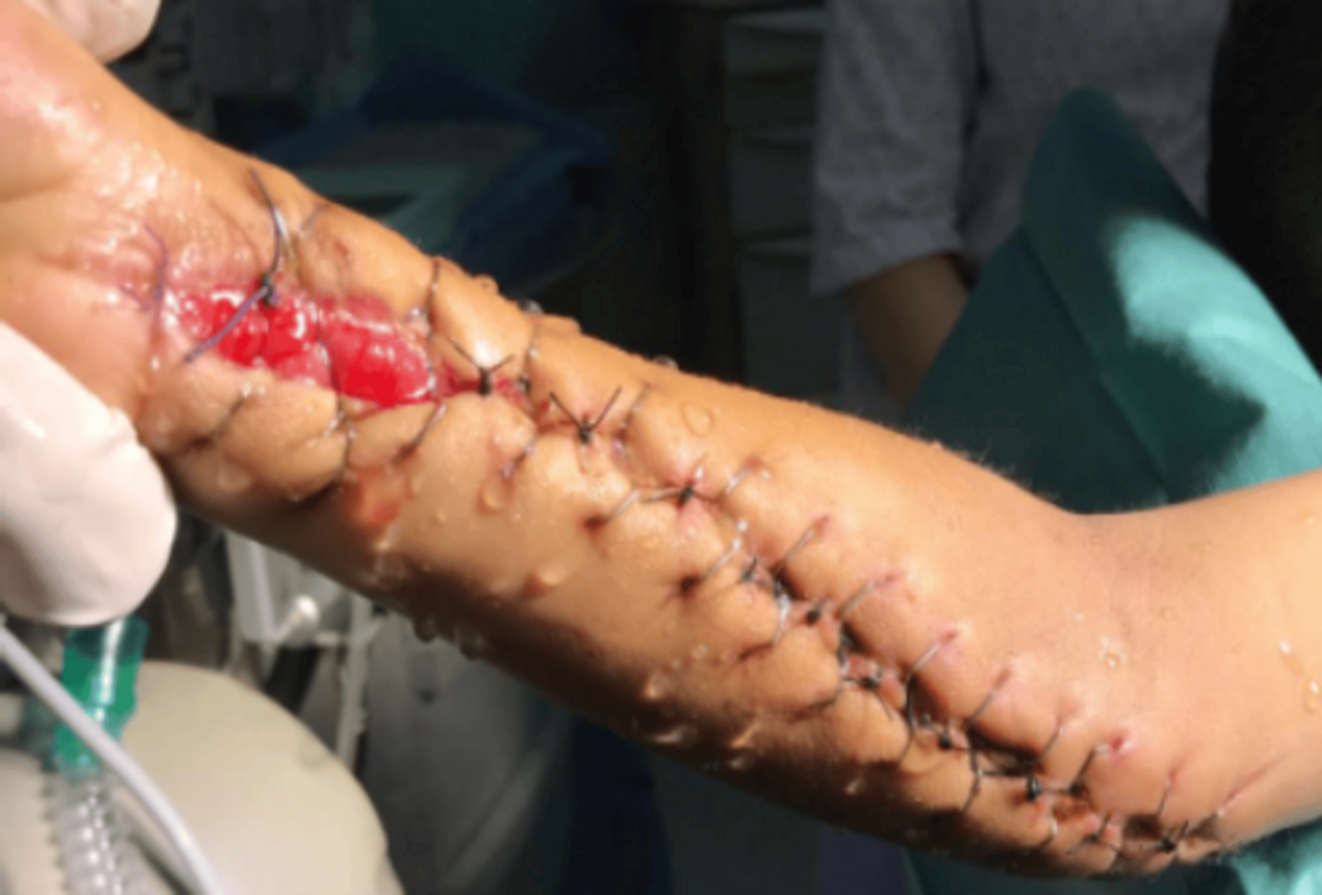
Aponeurotomy and progressive approximation

A favorable response to prednisone was observed in 17 patients. However, seven patients did not show improvement with oral corticosteroids, necessitating the introduction of second-line treatments. Immunosuppressants such as cyclosporine and cyclophosphamide were prescribed to three patients. One patient was treated with colchicine at a dose of 1 mg/kg/day due to persistent cutaneous signs and responded well to this treatment. Additionally, two patients underwent surgery: one for compartment syndrome of the upper limb (Figure 2) and the other for testicular torsion.

After an average follow-up period of one year, the short-term evolution of skin, joint, and digestive disorders was generally favorable, with symptoms regressing on average within 12 days. Patients with renal involvement showed significant improvement in treatment, with resolution of hematuria and proteinuria within three months. However, three patients experienced relapses: one developed moderate proteinuria, another progressed to nephrotic syndrome, and the third had a recurrence of rectal bleeding.

## Discussion

The annual incidence of RP is approximately 10 cases per 100,000 population [5], but no national study has yet assessed this incidence in our country. The disease mainly affects boys and children aged between 3 and 15, with a peak between the ages of four and seven [6-10]. Although RP is often more common in autumn and winter [7], some studies have also reported a predominance in spring and winter [11]. Our data confirm a significant increase in cases during the autumn and winter.

RP typically presents with an acute onset, with symptoms developing over a period of a few days to two or three weeks [6]. The disease encompasses a range of manifestations, including skin, joint, abdominal, digestive, and renal symptoms. Purpura, a crucial diagnostic feature, is present in 100% of cases and appears as red or brown petechial spots, developing over three to four days [8,12]. The purpura predominantly affects the lower limbs when the patient is in a standing position, with a higher prevalence on the buttocks and the back of the elbows when supine [6,10,13,14].

While facial lesions are rare [15], they were observed in 18% of cases in our series. Purpura generally resolves within one to two weeks, although recurrent flare-ups may persist for several months and are often associated with early rising. This highlights the importance of rest in the treatment regimen [3,6,7,12].

Joint involvement was noted in 100% of cases in our study, which is consistent with the higher end of the range reported in the literature (64% to 88.4%) [3,4,10,11,13,16]. Joint symptoms were inaugural in 22% of our cases compared to 26.4% reported elsewhere [17].

The large joints of the lower limbs, particularly the knees and ankles, are primarily affected, with a prevalence of 97% in our series. Upper joints, such as the wrists and elbows, are less frequently involved, affecting only 3% of patients in our study. Generally, joint symptoms resolve without sequelae within three to four days [7,12,13].

Digestive issues in RP primarily present as abdominal pain and vomiting. The presence of digestive hemorrhage, whether upper or lower, should raise concerns about potential complications that could impact the short-term prognosis. Major digestive complications include hemorrhage, acute intestinal intussusception, and perforation [18,19].

Renal involvement in RP varies widely, with reported rates ranging from 11.5% to 60.5% and an estimated prevalence of 35%, as reported by Wilhelm-Bals [20]. Renal manifestations may include hematuria, proteinuria, and, in some cases, nephrotic syndrome or renal failure.

Renal investigations in patients with RP typically reveal biological abnormalities. Microscopic hematuria is common, with its frequency varying across studies from 20% to 100% of cases [3,11,21,22]. Proteinuria, indicative of renal involvement, is often present and is generally benign if it remains below 0.50 g/24 hours. In our series, proteinuria was observed in up to 100% of cases, while other studies reported rates of approximately 92% [3,11,23]. Blood urea levels, which reflect glomerular filtration rate, are usually slightly elevated but generally transient, ranging from 0.70 g/L to 1 g/L [3,9,23].

Renal biopsy is crucial for accurate diagnosis, prognosis assessment, and therapeutic guidance [8,24-26]. It is indicated in cases of renal failure, persistent hypertension, or long-lasting renal abnormalities. The histological analysis identifies various types of glomerulonephritis, including mesangiopathic, segmental, and focal, as well as diffuse endocapillary and endo- and extracapillary; fibrous kidney; and IgA deposits, visible by immunofluorescence, which are commonly detected in renal biopsies and can be observed even when lesions are not apparent under light microscopy [12]. The most frequent glomerular abnormalities include segmental and focal glomerulonephritis, while membranoproliferative glomerulonephritis is rare and tends to have a less favorable prognosis. Electron microscopy often reveals deposits in the mesangium as well as changes in the pedicels and podocytes, even if the kidney appears normal on light microscopy.

The severity of the rash and the frequency of relapses are not directly correlated with the severity or frequency of renal damage. Therefore, it is crucial to extend renal monitoring, as kidney damage is detected at diagnosis in only 20% of cases. Young girls with renal involvement, even if mild and transient, are at increased risk of developing proteinuria or arterial hypertension during pregnancy compared to a control group [27]. This highlights the need for prolonged monitoring in children who have experienced RP.

Additionally, other organs may be involved [28], including the (1) ureters and bladder, with rare manifestations such as stenosing ureteritis, hemorrhagic cystitis, and hematomas; (2) testes, which are affected in 9% of cases in boys; (3) central nervous system, which is affected in 2% to 7% of cases with multifactorial pathology; (4) heart and pericardium, with conditions such as myocarditis and pericarditis; (5) lungs and pleura, with involvement occurring in less than 1% of cases; (6) muscles, including intramuscular hemorrhage and myositis; (7) parotid glands; (8) hepatosplenomegaly; and (9) cholecystitis.

These conditions are rare and generally have a favorable prognosis. The diagnosis of RP is primarily clinical, with additional investigations playing a limited role. Laboratory tests usually show normal or elevated levels of white blood cells and platelets, while anemia is uncommon except in cases of severe bleeding [29].

Hemostasis is generally normal, although there may be a transient increase in platelets [16], often associated with hypo-splenism and circulating immunocomplexes [30]. Factor VIII is frequently depleted, reflecting the severity of RP and indicating an increased risk of major complications if levels fall below 60% [12]. The inflammatory syndrome is typically moderate, with a slightly elevated sedimentation rate in 44% of cases and generally normal fibrinogen levels [12,30].

IgA levels are often elevated, and IgA-containing immune complexes are present in 30-50% of cases [12]. Immunological tests may show positivity for labeled C1q in 25% of cases [30]. Complement components are generally within normal ranges, although variations in C3, properdin, and C5 may be observed [15].

Imaging, particularly abdominal ultrasound, can confirm acute intestinal intussusception and detect hematomas [12].

Endoscopic examinations frequently reveal purpuric lesions, petechiae, and occasionally ulcerations. These examinations can show areas of purpura and congestion in the colon and rectum [31,32]. Treatment of RP is primarily symptomatic and tailored to the severity of the clinical manifestations. Generally, the condition tends to resolve spontaneously. Initial management typically includes bed rest, antispasmodics for abdominal pain, and antibiotic therapy if there is a concomitant bacterial infection [12,30].

While rest is crucial in the early stages, recent recommendations suggest gradual rising rather than prolonged bed rest [12]. Continuous low-flow enteral nutrition is often employed for managing digestive disorders, whereas parenteral nutrition is reserved for severe cases with complications [30].

Corticosteroid therapy can be beneficial, especially for managing testicular damage, but must be administered under strict supervision due to the risk of gastric ulceration [33]. Immunosuppressive drugs, such as azathioprine or cyclophosphamide, are reserved for severe nephropathy and should be guided by the histological results of renal biopsies [30,34]. Colchicine may be used for persistent cutaneous manifestations [35], while plasma exchange is considered an exceptional treatment for severe cases with significant digestive or renal complications [30].

Surgery is indicated only for serious complications, such as intestinal intussusception or perforation [9]. For nephropathy, regular monitoring is essential, including renal protective treatments and vigilant attention to moderate and severe cases. These cases require prolonged follow-up to detect potential long-term complications [3,15,30].

## Conclusions

RP is a well-defined pediatric condition often following an upper respiratory infection, marked by IgA deposits in affected tissues. Although typically benign, it can cause serious digestive and renal complications, necessitating close monitoring. Purpuric lesions in the bowel or testis can sometimes mimic acute abdomen or testicular torsion, underscoring the importance of background knowledge to avoid unnecessary surgeries. Corticosteroids are recommended for managing these complications, although their preventive efficacy is limited.
